# A planning approach for online adaptive proton therapy to cope with cone beam computed tomography inaccuracies^☆^

**DOI:** 10.1016/j.phro.2025.100752

**Published:** 2025-03-20

**Authors:** Michelle Oud, Sebastiaan Breedveld, Kelvin Ng Wei Siang, Roberto Cassetta, Steven Habraken, Zoltán Perkó, Ben Heijmen, Mischa Hoogeman

**Affiliations:** a Erasmus MC Cancer Institute, Department of Radiation Oncology, Rotterdam, the Netherlands; b HollandPTC, Delft, the Netherlands; c Varian, a Siemens Healthineers company, Baden, Switzerland; d Leiden University Medical Center, Department of Radiation Oncology, Leiden, the Netherlands; e Delft University of Technology, Department of Radiation Science and Technology, Delft, the Netherlands

**Keywords:** intensity modulated proton therapy (IMPT), cone beam computed tomography (CBCT), in-room CT, offline adaptive radiotherapy, online adaptive radiotherapy, head-and-neck cancer, inter-fraction motion

## Abstract

**Background and purpose:**

In online-adaptive proton therapy planning based on cone beam computed tomography (CBCT), CT number errors can pose challenges. We propose an approach for coping with CT number uncertainties by increasing range robustness settings (RRS) in online-adaptive planning. This was compared to our trigger-based offline (TB-Offline) adaptive approach, and to daily replanning using in-room CT-on-rails (CTOR).

**Material and methods:**

For 23 head-and-neck cancer patients, a CTOR and CBCT were acquired in a single fraction. CTOR contours were copied rigidly onto the CBCT. CBCT-based plans were generated with 3, 6, 8, 10, and 12 % RRS, each with 1 mm setup-RS, followed by a forward dose calculation on the reference CTOR. This was compared to dose distributions from our TB-Offline approach (3 mm/3% SRS/RRS), also recomputed on the CTOR. Coverage (voxelwise-minimum) of the primary clinical target volume (CTV_7000_) and elective lymph nodes (CTV_5425_) and grade ≥ II normal tissue complication probabilities were compared between strategies.

**Results:**

When going from RRS = 3 % to RRS = 10 %, the population 90th percentiles of CTV_5425_ V_94%_ improved from 89.6 % to 96.4 %, and CTV_7000_ V_94%_ from 92.8 % to 96.4 %. Substantial coverage loss (V_94%_<95 %) with CBCT-based online adaptive and RRS = 10 % was observed in 1/23 evaluated patients for CTV_7000_ and 2/23 for CTV_5425_. This was an improvement compared to 3/23 and 4/23 with TB-Offline. Moreover, for RRS = 10 % the average risk of xerostomia improved by 2.4 percentage point compared to TB-Offline.

**Conclusions:**

Robust optimization with increased range robustness settings effectively mitigated dose degradation from CT number errors in CBCT-based online-adaptive proton therapy.

## Highlights

• Iteratively reconstructed cone beam computed tomography images (CBCTs) were used.
• Ground truth images were CT-on-rails acquired within 3 min from the CBCT.
• Increased range robustness mitigated dose degradation from CT number errors.
• CBCT-based online adaptive outperformed our current offline adaptive approach.

## 1 Introduction

By daily adaptation of the treatment plan to the patient anatomy in treatment position, online re-optimization has the potential to improve target coverage and reduce the required margins or setup robustness settings (SRS), thereby reducing organ-at-risk (OAR) doses [1], [2], [3], [4], [5]. This is particularly relevant for intensity modulated proton therapy (IMPT), where the delivered dose is substantially more sensitive to variations in patient anatomy compared to photon therapy. Daily imaging is required for online adaptations. The use of cone beam computed tomography (CBCT) imaging for online-adaptive IMPT is favored primarily for its practicality over diagnostic CTs, for example in-room CT-on-rails (CTOR) or a near-room CT with a shuttle system. This is mostly due to its efficiency and availability as part of the standard daily clinical image-guided workflow. However, the lower CT number accuracy of CBCTs poses challenges for IMPT treatment planning [6], [7].

Recent advancements have improved CBCT-based dose computation accuracy for proton therapy. Methods using deformable image registration (DIR) of the planning-CT on the CBCT [8], [9], [10], scatter-correction of the CBCT raw projections using the deformed planning-CT [11], [12], a combined DIR and CT number artifact correction approach [13], [14], and methods using deep learning [15], [16], [17], [18], [19] have shown improved CT number accuracy. However, residual CT number errors in CBCT can still have a clinically relevant dosimetric impact for use in online-adaptive proton therapy, since even small variations in the CT numbers can result in substantial deviations in proton dose distributions [20], [21], [22].

Rather than focusing on enhancing CBCT quality, we propose an approach that integrates the residual CT number uncertainties in daily CBCT-based treatment planning via already applied robust IMPT optimization. In diagnostic CT-based treatment planning, robust optimization can account for uncertainties in relative stopping power (RSP) predictions using a range robustness setting (RRS) [23], [24]. Using increased RRSs in daily CBCT-based planning could possibly also compensate for RSP uncertainties caused by errors in CT numbers.

The aim of this study was to investigate whether the dosimetric impact of uncertainties in CT numbers of CBCTs can be mitigated by strongly increasing RRS, while still preserving a dosimetric benefit from online treatment plan adaptation to the daily anatomy, thereby allowing for a reduced SRS. The CBCT-based online adaptive strategy was compared to our clinical offline adaptive reference and to CT-on-rails-based online adaptive IMPT, representing the maximal dosimetric potential of online adaptations.

## 2 Materials and methods

### 2.1 Patient data and contours

Data from 23 head-and-neck cancer patients treated at Holland Proton Therapy Center (HollandPTC) in 2022 and 2023 who consented were included. A local Institutional medical review board waived the need to assess the protocol of the research database (approval number P18 053). Inclusion criteria were: 1) Raw CBCT data available and acquired correctly, 2) Complete clinical target volume (CTV) visible on the CBCT. For each patient, a single CTOR-CBCT pair was available.

The patient was moved in treatment position from the CTOR (SOMATOM Confidence CT, Siemens Healthineers, Erlangen, Germany) to the gantry (Varian ProBeam 4.0, Varian Medical Systems, Palo Alto, United States) equipped with a CBCT using a robotic couch. The CTOR and CBCT were acquired 3.1 ± 1.0 min apart while patients remained immobilized on a robotic couch. CBCTs were acquired with 125 kV tube voltage, and reconstructed with a grid size of 1.0x1.0x2.0 mm^3^, a field of view (FOV) of 521x521 mm^2^, and 104 axial slices. Iterative reconstruction [25] of CBCTs was performed using pre-clinical Varian software, which was tuned on phantom data and measurements acquired at our institute. CTORs were scheduled as standard part of treatment based on the availability of additional time for the treatment fraction. Depending on the protocol, one or multiple CTORs were acquired per patient. Only the first CTOR-CBCT pair was used for this study. Furthermore, for every patient, one out-of-room planning-CT and 0–3 regular repeat-CTs were available, out of which only one was used in this study (see Section 2.4). Planning-CTs and regular repeat-CTs were acquired with an out-of-room CT scanner (SOMATOM Definition Edge, Siemens Healthineers). Both in-room and out-of-room CTs were acquired with 120 kV tube voltage, and a resolution of 1.0x1.0x2.0 mm, a FOV of 500x500 mm. Detailed patient information on tumor site, target volumes, tumor stage, and baseline toxicities can be found in Supplementary Data, Table S.1.

Before each treatment fraction, the CBCT acquired at the gantry was registered to the CT used for planning, followed by a 6-D couch correction (translational shifts, pitch, roll, and yaw). For the purpose of this study, all CTORs and CBCTs were rigidly aligned to the out-of-room CT used for offline treatment plan generation. For the CBCTs, the registration acquired at the gantry was used. The clinically approved planning-CT contours were propagated to the CTOR. The CTV_7000_ was propagated rigidly, while the CTV_5425_ and OARs were propagated deformably. For one patient (Patient 5), CTOR contours were manually adjusted because a re-plan was triggered on this CTOR, and the CTV location changed. The contours of the CTOR were rigidly copied onto the CBCT.

### 2.2 Treatment planning and robust optimization

Patients were treated with a biologically-weighted dose of 70 Gy to the gross disease sites (CTV_7000_) and 54.25 Gy to the elective areas (CTV_5425_, with the CTV_7000_ as part of the CTV_5425_) in 35 fractions, assuming a constant relative biological effectiveness (RBE) of 1.1, with a target dose constraint of V_94%_>98 %. This had to be met in the voxel-wise minimum dose distribution (VoxMin, the composite of minimum dose values per voxel from the 29 scenarios) [26].

All treatment plans in this study, including the treatment plans on the CBCT, were generated with the in-house developed Erasmus-iCycle software for fully automated treatment planning [27], [28], [29], [30]. The software was tuned to generate treatment plans similar to clinical treatment plans. For all patients, a 6-beam setup was used (50, 100, 160, 200, 260, and 310 degrees angles). For the angles of 100 and 260 degrees, a couch rotation of 20 degrees was applied. In case the energy range of a beam was not sufficient to cover the superficial parts of the target, a range shifter of 34 mm (water equivalent thickness) was used. Metal implants and shoulders were delineated, and avoided during treatment planning by removing spots traveling through these areas from the optimization. Erasmus-iCycle used a wish-list to configure the multi-criterial optimization, which can be found in Supplementary Data, Table S.2.

Erasmus-iCycle used scenario-based mini-max robust optimization [1], [23], [24]. During robust optimization, dose requirements had to be met in each of the 29 optimized error scenarios: the nominal scenario, and 14 isocenter shifts combined with ± a range error. For range errors, robust optimization scenarios were generated by scaling the mass density [23], [24], [31]. Employed scaling factors are denoted as RRS. The magnitude of the isocenter shifts in the optimization scenarios are denoted as SRS. Only targets, ring structures around the targets and serial OARs were robustly optimized due to calculation time and memory restrictions. Other structures were optimized in the nominal scenario only.

### 2.3 Daily CBCT-based adaptive IMPT with increased RRS

In CT-based planning, several factors contribute to uncertainty in proton range, of which conversion of CT numbers into RSP is one of the main contributors [32], [33]. As commonly applied, we clinically use RRS = 3 % [34], [35], [36]. In CBCT-based treatment planning, additional uncertainties in CT numbers further contribute to RSP uncertainty. Although the source of these uncertainties differs from those in CT-based treatment planning, they also affect RSP uncertainty. In this study, we investigated robust CBCT-based adaptive planning with increased RRS (>3%) to both account for uncertainties in conversions of CT number to RSP and for uncertainties in CT numbers. In CBCT-based planning, the same CT-to-RSP curve was used as in CT-based planning.

### 2.4 Study design

In order to assess the effectiveness of robust CBCT-based online-adaptive treatment planning with increased RRS, 5 different treatment plans were generated per patient on the CBCT with RRS of 3 %, 6 %, 8 %, 10 %, and 12 %. From RRS = 6 %, we increased RRS in steps of 2 % until no further benefit in target coverage was observed. All treatment plans generated for the simulated online-adaptive strategies had 1 mm SRS, in line with literature and expected errors [4], [5], [37]. CBCT-based online-adaptive treatment planning was compared to two other treatment planning strategies: 1) A clinical reference: a strategy that mimics our current TB-Offline adaptive protocol. Robust treatment plans were generated using out-of-room CTs using SRS/RRS of 3 mm/3%, in line with our clinical settings. The out-of-room CT was either the planning-CT, or a repeat-CT in case an offline adaptation was triggered in clinical practice. Decisions to generate a new plan in the clinic were guided by visual inspection of sequential daily CBCTs and dose assessments for repeat-CTs. In this study, we adapted if the original clinical plan was adapted as well. 2) A reference for the full dosimetric potential of online-adaptive treatment planning: CTOR-based online-adaptive treatment planning, robustly optimized with 1 mm/3% SRS/RRS. For each evaluated strategy, dosimetric evaluations were performed through forward dose computations of the treatment plans on the reference CTOR, which was considered the ground truth. Table S.3 in the Supplementary Data summarizes the images and treatment plans used in the study for the different evaluated treatment planning strategies.

### 2.5 Evaluation

The mean absolute error (MAE) and mean errors (ME) in CT numbers were compared between the CBCTs and corresponding reference CTOR within the external (excluding air cavities with CT number < -960 HU). The mean and standard deviation over the population was reported. Coverage of the CTVs (V_94%_) was always evaluated in the VoxMin dose distribution with 1 mm/3% SRS/RRS [26] on the CTOR. The median and population 90th percentile were compared between strategies, where the population 90th percentile was calculated by linear interpolation between the 20th and 21st worst-performing patients out of 23. Doses to the OARs (constrictor muscles, oral cavity, parotids, and submandibular glands) were evaluated in the nominal scenario [38] on the CTOR, and converted to normal tissue complication probability (NTCP) through the models employed in the Dutch National Indication Protocol [39]. Statistical significance of dosimetric differences between treatment strategies were assessed using the Wilcoxon Signed-Rank test (α < 0.05).

## 3 Results

**Fig. 1:**
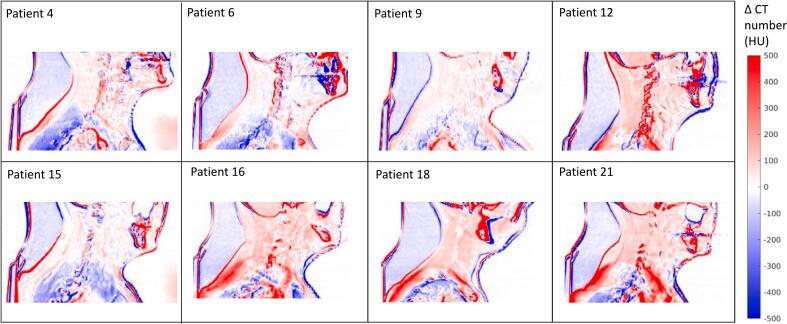
Differences in computed tomography (CT) numbers between the cone beam CT (CBCT) and reference CT-on-rails (CTOR) for eight randomly selected example patients. The color bar indicates differences in CT numbers, computed as CBCT-CTOR.

Fig. 1 exemplifies for randomly selected patients the difference in CT numbers of the CBCTs and reference CTOR. Relatively large differences can be seen especially around the bone, air, and caudal areas of the CBCTs. Moreover, differences also arose due to some intra-fraction motion between CTOR and CBCT, resulting in significant local CT number deviations. Additionally, for some patients, the CT numbers were in general elevated in the CBCT. The resulting MAE and ME of the CT numbers in the CBCTs compared to the CTOR were 145 ± 27 HU and 22 ± 45 HU, respectively.

**Fig. 2:**
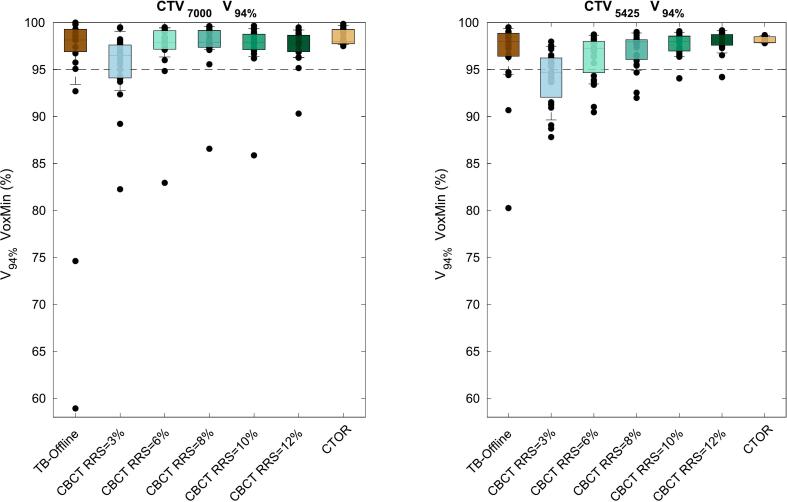
The V_94%_ in the voxelwise-minimum dose distribution (1 mm/3%, 29 scenarios, VoxMin) for the clinical target volumes (CTVs) CTV_7000_ (left) and CTV_5425_ (right), as calculated the computed tomography-on-rails (CTOR) for each of the 23 patients. Evaluated strategies were: our offline clinical trigger-based adaptation schedule (TB-Offline) with 3 mm setup robustness settings (SRS) and 3 % range robustness settings (RRS), treatment plans generated on the cone beam CT (CBCT) with 1 mm SRS and 3, 6, 8, 10, and 12 % RRS and reference treatment plans generated on the CTOR (1 mm/3% SRS/RRS). Every dot represents a patient, whiskers extent to the population 90 %. The dotted line represents the evaluated dose for negative outliers in coverage (<95 %, see Results section).

Fig. 2 shows that target coverage, as evaluated on the CTOR, in CBCT-based plans increased with increasing RRS, especially for CTV_5425_. For example, the population 90th percentile of CTV_5425_ V_94%_ in the VoxMin dose distribution improved from 89.6 % (RRS = 3 %), to 93.5 % (RRS = 6 %), to 94.9 % (RRS = 8 %), to 96.4 % (RRS = 10 %), to 96.8 % (RRS = 12 %). For CTV_7000_, the V_94%_ mainly improved going from RRS = 3 % to RRS = 6 %: 90th percentile improved 92.8 % to 96.3 %.

CBCT-based online-adaptive IMPT with RRS = 10 % resulted in fewer negative outliers in target coverage than our current TB-offline schedule: a coverage < 95 % was observed for 1/23 for CTV_7000_ and 2/23 CTV_5425_, while this was 3/23 and 4/23 with TB-offline. The median V_94%_ was similar: 97.9 % vs 98.1 % for the CTV_7000_ in favor of TB-offline (not significant), and 98.0 % for the CTV_5425_ for both strategies.

**Fig. 3:**
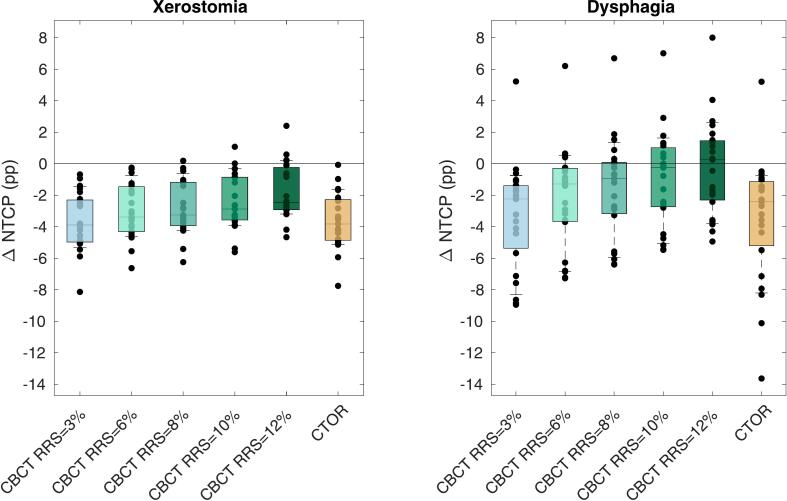
Differences in normal tissue complication probability (NTCP) for grade ≥ II xerostomia and dysphagia compared to our offline clinical trigger-based adaptation schedule (TB-Offline) for treatment plans generated online on cone beam computed tomography (CBCT) with 3, 6, 8, 10, and 12 % range robustness settings (RRS) and 1 mm setup RS (SRS), and for plans generated online on the reference CT-on-rails (CTOR) with 1 mm/3% SRS/RRS. NTCPs were calculated in percentage points (pp), and were based on the dose recalculated on the CTOR for each of the 23 patients. Negative values are in favor of the online adaptation strategies. Whiskers extent to the population 90 %. Every dot represents a patient.

Fig. 3 shows the NTCP difference between TB-Offline and the CBCT-based plans. Online CBCT plans had overall reduced xerostomia and dysphagia NTCP compared to TB-Offline, but the gain decreased with increasing RRS. For RRS = 10 %, mean improvement in the risk of xerostomia was still 2.4 ± 1.7 percentage point (pp) (mean ± standard deviation, p < 0.001), while differences in the risk of dysphagia were non-significant. One patient (Patient 12) had a significant increase in risk of dysphagia (7.0 pp with RRS = 10 %), but the target coverage was strongly improved in CBCT-based online-adaptive approach with RRS = 10 % (V_94%_ of 96.2 % and 98.9 % for the CTV_7000_ and CTV_5425_, compared to 74.6 % and 90.7 % for TB-Offline).

The reference CTOR-based treatment plans resulted in average reductions of 3.6 ± 1.8 pp for xerostomia and 3.5 ± 3.9 pp for dysphagia compared to TB-Offline. This means that with CBCT-based online-adaptive planning using RRS = 10 %, (where xerostomia reduction was 2.4 pp) 67 % of the potential maximum xerostomia reduction was achieved.

Compared to TB-Offline, mean doses across all investigated OARs and RRS, except the oral cavity, were improved in the CBCT-based treatment plans, with p-values below 0.004 across all OARs and treatment plans (Supplementary Data, Fig. S.1).

## 4 Discussion

This study investigated robust optimization in daily CBCT-based adaptive planning to mitigate dose degradation as a result of errors in CT numbers. Increased RRS effectively mitigated dose degradation resulting from CT number errors in CBCTs. When increasing from RRS = 3 % to RRS = 10 %, the population 90th percentiles of CTV_5425_ V_94%_ improved from 89.6 % to 96.4 %, and CTV_7000_ V_94%_ from 92.8 % to 96.4 %. Compared to our clinical trigger-based offline adaptive approach (TB-Offline) with unavoidably larger setup robustness settings to account for inter-fraction motion, the CBCT-based online-adaptive approach with 10 % RRS largely reduced extreme target coverage losses, achieved similar median target coverage, while also reducing the risk of xerostomia significantly.

Even with a RRS as large as 10 %, the mean improvement in the risk of xerostomia was 2.4 pp compared to TB-Offline, which was 67 % of the total maximal potential NTCP reduction that could be achieved with daily CTOR-based treatment planning. These positive NTCP results are a consequence of the reduction in setup robustness settings allowed in online-adaptive treatment planning. Even with 1 mm/12 % SRS/RRS, the V_10Gy_ and the total number of monitor units of the CBCT-based plans remained significantly lower compared to TB-Offline with 3 mm/3% RS. This is probably a result of the fact that range robustness results in a 1D expansion of the treated volume in the beam direction. This is feasible at lower dosimetric costs compared to the gain resulting from lowering the setup robustness setting, which is a 3D reduction of the treated volume. This was also previously observed in Van de Water et al. [1], although not for range robustness settings up to 12 %. Moreover, the expansion of the volume in the beam direction and the multiple beam angles used could possibly result in a redistributed dose in such a way that the surplus was not delivered in critical OARs.

Previous studies reported improved dose metrics with CBCT-based IMPT compared to non-adaptive approaches [12], [14], [40]. An advantage of our study compared to these studies is the use of realistically lower setup robustness settings for the online-adaptive approach, the use of a reference CT acquired only minutes before the CBCT while patients remained fixated on the couch, the robustness analyses on the reference CT, and the comparison to our clinical offline adaptive schedule.

Compared to previous studies on the use of CBCTs for proton dose computations, our work incorporates several differences. Only Xu et al. [40] and Kaushik et al. [14] used robust treatment plan optimization in this context. However, they only took regular CT-based uncertainties in the conversion from CT numbers to RSP into account. Compared to other studies, the MAE and ME of our CBCTs were found to be larger than in other studies [11], [21], [41], [15], [16], [17], [18], [19]. This difference could be caused by the decision not to deform the CBCT to the CT, which we regarded as ground truth. Some intra-fraction motion may have occurred resulting in increased MAE and ME. Additionally, it can be caused by variation in CT numbers between CBCT scanners. An advantage of the employed CBCTs in this study is that the reconstruction software is expected to be clinically available in the near future, and no artificially generated CT is expected to be required. In the future, CT number accuracy of CBCTs is expected to further improve. Therefore, it is likely that CBCTs will be used for online-adaptive IMPT. Our approach will stay relevant to mitigate the impact of remaining CT number uncertainties.

In this study, we used a CT-based calibration curve enabling us to demonstrate the feasibility of our approach. In a clinical setting, our approach should be accompanied by a CBCT-specific CT-to-RSP calibration curve, generated based on phantoms with known tissue-equivalent materials, directly relating CT-numbers of CBCTs to RSP. Such a curve is challenging to acquire due to the CT-number errors in CBCTs, but it is feasible to obtain an improved calibration curve. With a CBCT-specific calibration curve, or in general improved CBCT quality, the required RRS might be reduced.

A limitation of this study is that two patients (Patient 10 and 18) in the dataset were presumably repositioned after acquiring the CTOR and CBCT because of poor alignment between CBCT and planning-CT. Despite this, target coverage in the TB-Offline strategy remained relatively high for these patients (V_94%_ > 97.7 % for both CTVs). Furthermore, additional contouring uncertainties on CBCTs were not taken into account. Additionally, motion between the CTOR and CBCT occurred, despite the use of a robotic couch. This contributed to significant deviations in local CT numbers, resulting in an overestimation of actual CT number errors in the CBCTs. Another limitation is that we only evaluated per-fraction doses instead of accumulated doses. Next, we used full re-optimization instead of a fast online re-optimization algorithm for treatment plan generation on the CBCTs and CTOR to avoid any bias related to differences in optimization algorithms. However, our online re-optimization algorithm has achieved comparable quality to fully re-optimized treatment plans [42]. Furthermore, fast online adaptation strategies are available [5], [43], [44], [45], [46]. For example, online re-optimization times for head-and-neck cancer patients using our algorithm were 3.4 min on average.

In conclusion, robust optimization in daily online CBCT-based planning using increased range robustness settings can effectively mitigate dose degradation resulting from CT number errors in CBCTs. Even for the relatively large CT number errors in our CBCT dataset, the online plans overall outperformed our current clinical offline adaptive approach in terms of mitigation of extreme target coverage losses and toxicity risks. With the expected introduction of CBCT-based online-adaptive treatment planning for head-and-neck IMPT, this clinically available method can ensure robustness against (residual) CT number errors.

## Declaration of competing interest

The authors declare that they have no known competing financial interests or personal relationships that could have appeared to influence the work reported in this paper.
