# Supplementary material for: A planning approach for online adaptive proton therapy to cope with cone beam computed tomography inaccuracies
Source: Phys Imaging Radiat Oncol. 2025 Mar 20;34:100752. doi: 10.1016/j.phro.2025.100752 (PMC11984568; doi:10.1016/j.phro.2025.100752)
Supplement: Supplementary Data 1 [file mmc1.pdf]

Supplementary Data

Table S.1 - Patient characteristics and treatment information. Abbreviations: Clinical Target Volume (CTV), Tumor (T), Node (N) stage in correspondence to [1]. Common Terminology Criteria for Adverse Events (CTCAE).

1. Brierley, J.D., M.K. Gospodarowicz, and C. Wittekind, TNM classification of malignant tumours. 2017: John Wiley & Sons.

	TUMOR SITE	VOLUME CTV_{7000}, CTV_{5425} ON PLANNING-CT (CM^3)	T	N	BASELINE XEROSTOMIA (0–3 CTCAE)	BASELINE DYSPHAGIA (0–5 CTCAE)
**1**	Oropharynx	54.2, 236.8	1	1	0	0
**2**	Oropharynx	53.3, 214.4	2	0	0	1
**3**	Oropharynx	49.6, 275.8	3	0	0	0
**4**	Hypopharynx	60.1, 308.6	3	2	0	0
**5**	Oropharynx	116.1, 362.7	2	1	0	1
**6**	Oropharynx	129.5, 385.3	3	1	0	0
**7**	Nasopharynx	28.2, 357.0	1	1	0	0
**8**	Oropharynx	86.4, 269.1	2	2c	0	0
**9**	Oropharynx	38.3, 235.5	2	0	0	2
**10**	Oropharynx	122.5, 302.2	4a	1	1	2
**11**	Oropharynx	92.6, 413.2	4	1	0	2
**12**	Oropharynx	148.9, 404.6	4a	2b	0	0
**13**	Oropharynx	222.6, 488.6	4	2	0	2
**14**	Oropharynx	159.4, 451.7	1	2b	0	1
**15**	Oropharynx	63.2, 226.0	1	2b	0	1
**16**	Oropharynx	128.3, 343.6	2	2b	1	0
**17**	Oropharynx	8.2, 308.0	0	1	0	0
**18**	Oropharynx	96.6, 214.3	2	1	0	0
**19**	Nasopharynx	248.7, 574.2	1	1	0	0
**20**	Oropharynx	56.1, 328.8	2	1	0	0
**21**	Oropharynx	25.1, 181.3	2	0	0	0
**22**	Oropharynx	81.2, 302.7	4a	1	0	0
**23**	Oropharynx	97.5, 272.3	1	2a	0	0

Table S.2 - Erasmus-iCycle wish-list for intensity modulated proton therapy for head and neck cancer patients. Abbreviations: Clinical Target Volume (CTV). $CTV_{5425\_intermediate\_10mm}$ was the part of the CTV_{5425} excluding the CTV_{7000} and within a distance of 10 mm from the CTV_{7000}. $CTV_{5425\_shrunk\_10mm}$ was the CTV_{5425} excluding CTV_{7000} and $CTV_{5425\_intermediate\_10mm}$. Serial organs-at-risk and ring structures were optimized in 9 scenarios (nominal scenario, 6 isocenter shifts, and 2 range shifts) in order to minimize optimization time.

* Objective was lower (74.9 Gy) in CBCT-based treatment plans to obtain similar maximum dose. ** Erasmus-iCycle specific monitor units (MU), corresponds to maximum MU per spot for head and neck treatment plans at Holland Proton Therapy Center.

**CONSTRAINTS**					
	**Structure**	**Function**	**Constraint**		**Robust**
	CTV_{7000}	Minimum	67.2 Gy		29 scenarios
	$CTV_{5425\_shrunk\_10mm}$	Minimum	52.4 Gy		29 scenarios
	$CTV_{Intermediate\_10mm}$	Minimum	52.4 Gy		29 scenarios
	CTV	Maximum / beam	47.0 Gy		29 scenarios
**OBJECTIVES**					
**PRIORITY**	**Structure**	**Function**	**Objective**	**Goal**	**Robust**
**1**	CTV_{7000}	↓ maximum	77.0 Gy*	77.0 Gy*	29 scenarios
**1**	$CTV_{5425\_shrunk\_10mm}$	↓ maximum	70.7 Gy	70.7 Gy	29 scenarios
**1**	$CTV_{intermediate\_10mm}$	↓ maximum	60.8 Gy	58.6 Gy	29 scenarios
**2**	CTV ring 0-10 mm	↓ maximum	64.0 Gy	59.8 Gy	9 scenarios
**2**	CTV ring 10-15 mm	↓ maximum	54.3 Gy	54.3 Gy	No
**3**	MU	↓ maximum	35.5** MU	35.5** MU	No
4	Mandible	↓ maximum	70.0 Gy	68.0 Gy	9 scenarios
**5**	Brainstem	↓ maximum	35.0 Gy	10.0 Gy	9 scenarios
**5**	Spinal cord	↓ maximum	35.0 Gy	10.0 Gy	9 scenarios
**6**	Cochlea	↓ mean	30.0 Gy	5.0 Gy	No
**7**	Optic nerve	↓ maximum	30.0 Gy	5.0 Gy	9 scenarios
**7**	Optic chiasm	↓ maximum	30.0 Gy	5.0 Gy	9 scenarios
**8**	Lens	↓ maximum	5.0 Gy	1.0 Gy	No
**8**	Eye	↓ mean	30 Gy	1.0 Gy	No
**9**	Brain	↓ maximum	1.0 Gy	1.0 Gy	No
**10**	Parotid	↓ mean	1.0 Gy	1.0 Gy	No
**11**	Submandibular gland	↓ mean	1.0 Gy	1.0 Gy	No
**12**	Constrictor muscle superior	↓ mean	1.0 Gy	1.0 Gy	No
**12**	Constrictor muscle medial	↓ mean	1.0 Gy	1.0 Gy	No
**13**	Constrictor muscle inferior	↓ mean	1.0 Gy	1.0 Gy	No
**13**	Larynx supra glottis	↓ mean	1.0 Gy	1.0 Gy	No
**13**	Glottic area	↓ mean	1.0 Gy	1.0 Gy	No
**14**	Cricopharyngeus	↓ mean	1.0 Gy	1.0 Gy	No
**15**	CTV Ring 0-10 mm	↓ mean	1.0 Gy	1.0 Gy	9 scenarios
**15**	CTV Ring 10-25 mm	↓ mean	1.0 Gy	1.0 Gy	No
**16**	MU	↓ mean	1.0 MU	1.0 MU	No

Table S.3 – Images and treatment plans used in the study for the different evaluated treatment planning strategies. Abbreviations: Trigger-based offline adaptive strategy (TB-offline), cone beam computed tomography (CBCT), setup robustness settings (SRS), range robustness settings (RSR).

***** The CT used for treatment plan generation in the TB-Offline strategy was either the planning-CT, or a repeat-CT in case an offline adaptation was triggered in clinical practice.

IMAGE	USED FOR EVALUATION OF TREATMENT PLANNING STRATEGY	WHEN ACQUIRED	TREATMENT PLANS ROBUSTNESS SETTINGS (SRS/RRS)
**PLANNING-CT***	TB-Offline	Before treatment	• 3 mm/3%
**REPEAT-CT***	TB-Offline	During treatment, ≥ 6 days before CBCT and in-room-CT	• 3 mm/3%
**IN-ROOM CT**	In-room CT-based online adaptive	During treatment	• 1 mm/3%
**CBCT**	CBCT-based online adaptive	During treatment, acquired 3.1 ± 1.0 minutes after in-room CT while patient remained immobilized on a robotic couch	• 1 mm/3%<br>• 1 mm/6%<br>• 1 mm/8%<br>• 1 mm/10%<br>• 1 mm/12%

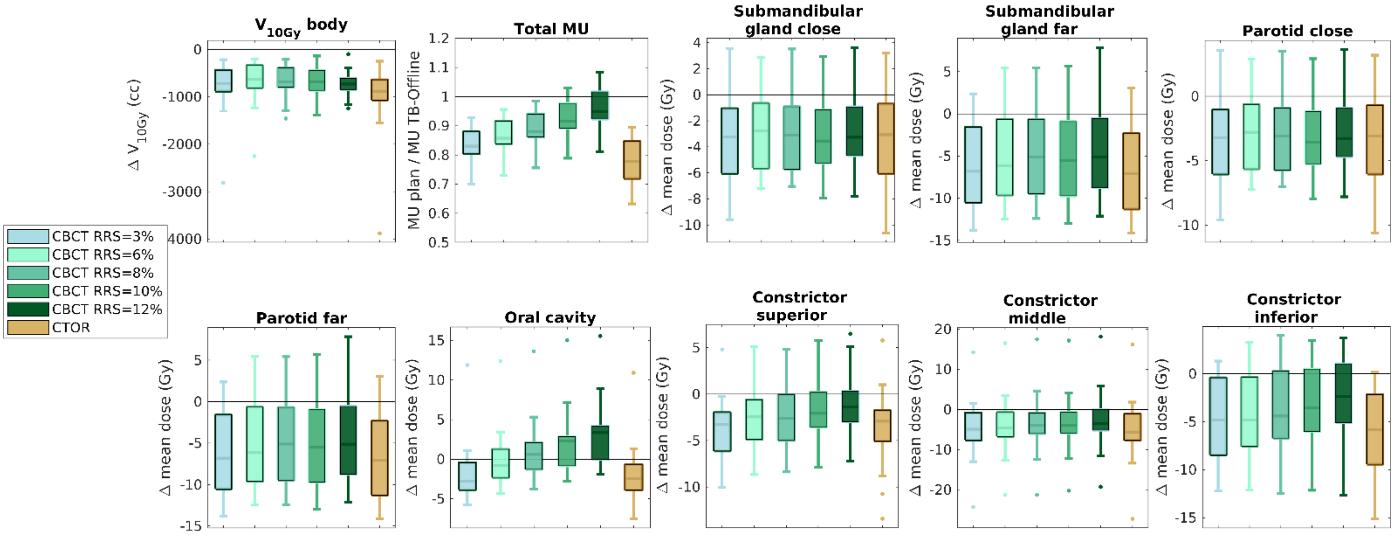

Figure S.1 – The differences in V_{10Gy}, total monitor units (MU), and mean dose to the OARs in the cone-beam CT (CBCT) and CT-on-rails (CTOR)-based online adaptive treatment plans, compared to the treatment plan in the trigger-based offline strategy (TB-Offline), as calculated on the CTOR. The dose to the submandibular glands and parotids was split into the gland receiving the highest (close) and lowest (far) dose. The corresponding p-values were below 0.004 across all organs-at-risk (OARs) and treatment plans. The V_{10Gy} and total number of MUs were significantly improved in the CBCT-based adaptive schedule compared to our current TB-offline schedule ($p<0.011$ for all treatment plans).
